# 1,3-Bis(chloro­meth­yl)-2-methyl-5-nitro­benzene

**DOI:** 10.1107/S1600536808007290

**Published:** 2008-03-20

**Authors:** Chang-Lun Shao, Chunyuan Li, Zhen Liu, Mei-Yan Wei, Chang-Yun Wang

**Affiliations:** a School of Medicine and Pharmacy, Ocean University of China, Qingdao, Shandong 266003, People’s Republic of China; b College of Science, South China Agricultural University, Guangzhou, Guangdong 510642, People’s Republic of China; c College of Chemistry and Chemical Engineering, Luoyang Normal University, Luoyang, Henan 471022, People’s Republic of China; d School of Pharmacy, Guangdong Medical College, Dongguan, Guangdong 523808, People’s Republic of China

## Abstract

The title compound, C_9_H_9_Cl_2_NO_2_, is a natural product isolated from the endophytic fungus No. B77 of the mangrove tree from the South China Sea coast. In the crystal structure, the mol­ecules lie on twofold axes and form offset stacks through face-to-face π–π inter­actions. Adjacent mol­ecules in each stack are related by a centre of inversion and have an inter­planar separation of 3.53 (1) Å, with a centroid–centroid distance of 3.76 (1) Å. Between stacks, there are C—H⋯O inter­actions to the nitro groups and Cl⋯Cl contacts of 3.462 (1) Å.

## Related literature

For related literature, see: McBee (1951 ▶).
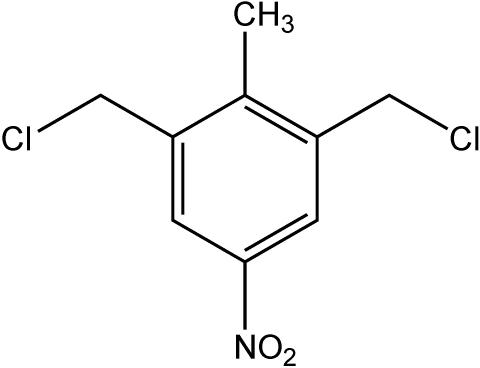


## Experimental

### 

#### Crystal data

C_9_H_9_Cl_2_NO_2_
*M* *_r_* = 234.07
Monoclinic, 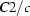

*a* = 8.921 (3) Å
*b* = 16.141 (6) Å
*c* = 7.511 (3) Å
β = 111.929 (6)°
*V* = 1003.3 (6) Å^3^
*Z* = 4
Mo *K*α radiation
μ = 0.62 mm^−1^
*T* = 273 (2) K
0.47 × 0.38 × 0.18 mm

#### Data collection

Bruker SMART CCD diffractometer
Absorption correction: multi-scan (*SADABS*; Bruker, 2001 ▶) *T*_min_ = 0.760, *T*_max_ = 0.897
2900 measured reflections
1113 independent reflections
976 reflections with *I* > 2σ(*I*)
*R*_int_ = 0.017

#### Refinement

*R*[*F* ^2^ > 2σ(*F* ^2^)] = 0.035
*wR*(*F* ^2^) = 0.106
*S* = 1.06
1102 reflections
66 parameters
H-atom parameters constrained
Δρ_max_ = 0.34 e Å^−3^
Δρ_min_ = −0.39 e Å^−3^

### 

Data collection: *SMART* (Bruker, 1997 ▶); cell refinement: *SAINT* (Bruker, 2001 ▶); data reduction: *SAINT*; program(s) used to solve structure: *SHELXS97* (Sheldrick, 2008 ▶); program(s) used to refine structure: *SHELXL97* (Sheldrick, 2008 ▶); molecular graphics: *SHELXTL* (Sheldrick, 2008 ▶); software used to prepare material for publication: *SHELXTL*.

## Supplementary Material

Crystal structure: contains datablocks global, I. DOI: 10.1107/S1600536808007290/bi2284sup1.cif

Structure factors: contains datablocks I. DOI: 10.1107/S1600536808007290/bi2284Isup2.hkl

Additional supplementary materials:  crystallographic information; 3D view; checkCIF report

## Figures and Tables

**Table 1:** Hydrogen-bond geometry (Å, °)

*D*—H⋯*A*	*D*—H	H⋯*A*	*D*⋯*A*	*D*—H⋯*A*
C6—H6*A*⋯O1^i^	0.97	2.66	3.427 (3)	136

Symmetry code: (i) 
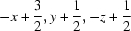
.

**Fig. 1.**
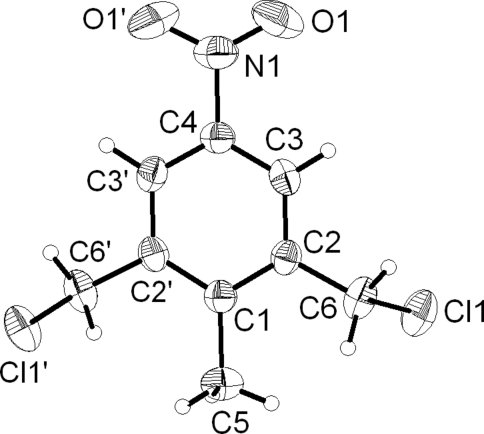
The molecular structure with displacement ellipsoids at 30% probability for non-H atoms. Primed atoms are generated by the symmetry operator: -x + 2, y, -z + 1/2.

**Fig. 2.**
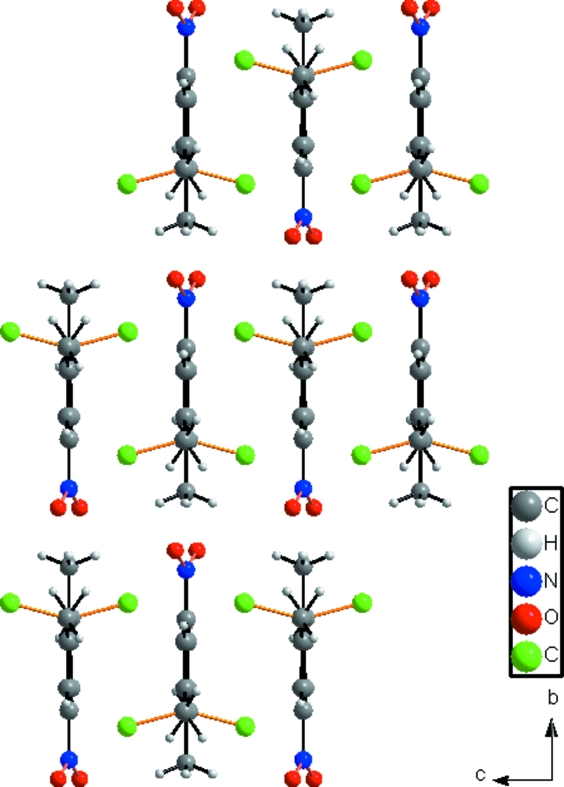
View of the packing along the normal to the bc-plane.

## supplementary crystallographic information

### Comment

The title compound was isolated from the endophytic fungus No.B77 from the mangrove tree from the South China Sea coast. As far as we know, this compound has not been reported previously as a natural product, but only as a synthetic compound (Mcbee, 1951). The molecules lie on crystallographic twofold axes (Fig. 1) and form offset π···π stacks (Fig. 2).

### Experimental

A strain of fungus (No. B77) was deposited in the Department of Applied Chemistry, Zhongshan University, Guangzhou, P. *R*. China. Culture conditions: GYT medium (glucose 10 g/*L*, peptone 2 g/*L*, yeast extract 1 g/*L*, NaCl 2.5 g/*L*) incubated at 298 K for 30 d. For the extraction and separation of the metabolite, the cultures (130 *L*) were filtered through cheesecloth, the filtrate was concentrated to 3 *L* below 323 K, then extracted three times by shaking with an equal volume of ethyl acetate. The extract was evaporated under reduced pressure and the combined organic extracts were subjected to silica-gel column chromatography, eluting with petroleum ether/ethyl acetate. Crystals of the title compound were obtained by evaporation of a methanol solution.

### Refinement

H atoms were positioned geometrically and treated as riding, with C—H = 0.93 (aromatic CH), 0.96 (methyl CH_3_) or 0.97 Å (methylene CH_2_), and with *U*_iso_(H) = 1.2*U*_eq_(CH) or 1.5*U*_eq_(CH_3_, CH_2_).

### Crystal data

**Table:**

C_9_H_9_Cl_2_NO_2_	*F*_000_ = 480
*M**_r_* = 234.07	*D*_x_ = 1.550 Mg m^−^^3^
Monoclinic, *C*2/*c*	Mo *K*α radiation λ = 0.71073 Å
Hall symbol: -C 2yc	Cell parameters from 711 reflections
*a* = 8.921 (3) Å	θ = 2.5–27.1º
*b* = 16.141 (6) Å	µ = 0.62 mm^−^^1^
*c* = 7.511 (3) Å	*T* = 273 (2) K
β = 111.929 (6)º	Block, colourless
*V* = 1003.3 (6) Å^3^	0.47 × 0.38 × 0.18 mm
*Z* = 4	

### Data collection

**Table:**

Bruker SMART CCD diffractometer	1113 independent reflections
Radiation source: fine-focus sealed tube	976 reflections with *I* > 2σ(*I*)
Monochromator: graphite	*R*_int_ = 0.017
*T* = 273(2) K	θ_max_ = 27.1º
φ and ω scans	θ_min_ = 2.5º
Absorption correction: multi-scan(SADABS; Bruker, 2001)	*h* = −11→9
*T*_min_ = 0.760, *T*_max_ = 0.897	*k* = −20→19
2900 measured reflections	*l* = −9→9

### Refinement

**Table:**

Refinement on *F*^2^	Secondary atom site location: difference Fourier map
Least-squares matrix: full	Hydrogen site location: inferred from neighbouring sites
*R*[*F*^2^ > 2σ(*F*^2^)] = 0.035	H-atom parameters constrained
*wR*(*F*^2^) = 0.106	*w* = 1/[σ^2^(*F*_o_^2^) + (0.0614*P*)^2^ + 0.6055*P*] where *P* = (*F*_o_^2^ + 2*F*_c_^2^)/3
*S* = 1.06	(Δ/σ)_max_ < 0.001
1102 reflections	Δρ_max_ = 0.34 e Å^−^^3^
66 parameters	Δρ_min_ = −0.39 e Å^−^^3^
Primary atom site location: structure-invariant direct methods	Extinction correction: none

### Special details

**Table:**

Geometry. All e.s.d.'s (except the e.s.d. in the dihedral angle between two l.s. planes) are estimated using the full covariance matrix. The cell e.s.d.'s are taken into account individually in the estimation of e.s.d.'s in distances, angles and torsion angles; correlations between e.s.d.'s in cell parameters are only used when they are defined by crystal symmetry. An approximate (isotropic) treatment of cell e.s.d.'s is used for estimating e.s.d.'s involving l.s. planes.
Refinement. Refinement of *F*^2^ against ALL reflections. The weighted *R*-factor *wR* and goodness of fit *S* are based on *F*^2^, conventional *R*-factors *R* are based on *F*, with *F* set to zero for negative *F*^2^. The threshold expression of *F*^2^ > σ(*F*^2^) is used only for calculating *R*-factors(gt) *etc*. and is not relevant to the choice of reflections for refinement. *R*-factors based on *F*^2^ are statistically about twice as large as those based on *F*, and *R*- factors based on ALL data will be even larger.

### Fractional atomic coordinates and isotropic or equivalent isotropic displacement parameters (Å^2^)

**Table:**

	*x*	*y*	*z*	*U*_iso_*/*U*_eq_	Occ. (<1)
Cl1	0.58497 (5)	0.11383 (3)	−0.00170 (7)	0.0545 (2)	
N1	1.0000	−0.17528 (13)	0.2500	0.0507 (5)	
C3	0.86254 (17)	−0.04274 (9)	0.2433 (2)	0.0347 (3)	
H3A	0.7713	−0.0721	0.2379	0.042*	
C1	1.0000	0.08774 (13)	0.2500	0.0332 (4)	
C2	0.86253 (17)	0.04352 (9)	0.2446 (2)	0.0328 (3)	
C4	1.0000	−0.08388 (13)	0.2500	0.0347 (5)	
O1	0.8703 (2)	−0.21053 (9)	0.2040 (3)	0.0840 (6)	
C5	1.0000	0.18142 (15)	0.2500	0.0528 (6)	
H5A	0.8974	0.2012	0.2461	0.079*	0.50
H5B	1.0186	0.2012	0.1395	0.079*	0.50
H5C	1.0840	0.2012	0.3644	0.079*	0.50
C6	0.71110 (19)	0.08572 (12)	0.2402 (2)	0.0438 (4)	
H6A	0.7393	0.1351	0.3195	0.053*	
H6B	0.6520	0.0489	0.2925	0.053*	

### Atomic displacement parameters (Å^2^)

**Table:**

	*U*^11^	*U*^22^	*U*^33^	*U*^12^	*U*^13^	*U*^23^
Cl1	0.0409 (3)	0.0665 (4)	0.0483 (3)	0.01565 (19)	0.0076 (2)	0.00775 (19)
N1	0.0643 (14)	0.0341 (10)	0.0476 (12)	0.000	0.0139 (10)	0.000
C3	0.0296 (7)	0.0409 (8)	0.0314 (7)	−0.0052 (6)	0.0088 (6)	0.0017 (6)
C1	0.0345 (10)	0.0339 (10)	0.0289 (10)	0.000	0.0092 (8)	0.000
C2	0.0292 (7)	0.0403 (8)	0.0276 (7)	0.0035 (6)	0.0091 (6)	0.0004 (5)
C4	0.0394 (11)	0.0311 (10)	0.0300 (10)	0.000	0.0087 (8)	0.000
O1	0.0821 (12)	0.0432 (8)	0.1149 (15)	−0.0220 (8)	0.0233 (11)	−0.0013 (9)
C5	0.0587 (16)	0.0335 (12)	0.0644 (17)	0.000	0.0209 (13)	0.000
C6	0.0346 (8)	0.0570 (10)	0.0398 (8)	0.0109 (7)	0.0139 (7)	0.0025 (7)

### Geometric parameters (Å, °)

**Table:**

Cl1—C6	1.8017 (18)	C1—C5	1.512 (3)
N1—O1	1.2178 (19)	C2—C6	1.502 (2)
N1—C4	1.475 (3)	C5—H5A	0.960
C3—C4	1.3787 (19)	C5—H5B	0.960
C3—C2	1.392 (2)	C5—H5C	0.960
C3—H3A	0.930	C6—H6A	0.970
C1—C2	1.4064 (18)	C6—H6B	0.970
			
O1—N1—O1^i^	124.3 (2)	C3—C4—N1	118.79 (10)
O1—N1—C4	117.85 (12)	C1—C5—H5A	109.5
O1^i^—N1—C4	117.85 (12)	C1—C5—H5B	109.5
C4—C3—C2	118.94 (14)	H5A—C5—H5B	109.5
C4—C3—H3A	120.5	C1—C5—H5C	109.5
C2—C3—H3A	120.5	H5A—C5—H5C	109.5
C2—C1—C2^i^	119.01 (19)	H5B—C5—H5C	109.5
C2—C1—C5	120.50 (10)	C2—C6—Cl1	110.75 (11)
C2^i^—C1—C5	120.50 (10)	C2—C6—H6A	109.5
C3—C2—C1	120.34 (13)	Cl1—C6—H6A	109.5
C3—C2—C6	117.12 (14)	C2—C6—H6B	109.5
C1—C2—C6	122.53 (15)	Cl1—C6—H6B	109.5
C3^i^—C4—C3	122.4 (2)	H6A—C6—H6B	108.1
C3^i^—C4—N1	118.79 (10)		
			
C4—C3—C2—C1	0.91 (19)	C2—C3—C4—N1	179.54 (9)
C4—C3—C2—C6	−179.08 (11)	O1—N1—C4—C3^i^	−164.92 (14)
C2^i^—C1—C2—C3	−0.46 (10)	O1^i^—N1—C4—C3^i^	15.08 (14)
C5—C1—C2—C3	179.54 (10)	O1—N1—C4—C3	15.08 (14)
C2^i^—C1—C2—C6	179.53 (15)	O1^i^—N1—C4—C3	−164.92 (14)
C5—C1—C2—C6	−0.47 (15)	C3—C2—C6—Cl1	−95.84 (15)
C2—C3—C4—C3^i^	−0.46 (9)	C1—C2—C6—Cl1	84.18 (15)

Symmetry codes: (i) −*x*+2, *y*, −*z*+1/2.

### Hydrogen-bond geometry (Å, °)

**Table:**

*D*—H···*A*	*D*—H	H···*A*	*D*···*A*	*D*—H···*A*
C6—H6A···O1^ii^	0.97	2.66	3.427 (3)	136

Symmetry codes: (ii) −*x*+3/2, *y*+1/2, −*z*+1/2.
